# Terpene Derivatives as a Potential Agent against Antimicrobial Resistance (AMR) Pathogens

**DOI:** 10.3390/molecules24142631

**Published:** 2019-07-19

**Authors:** Nik Amirah Mahizan, Shun-Kai Yang, Chew-Li Moo, Adelene Ai-Lian Song, Chou-Min Chong, Chun-Wie Chong, Aisha Abushelaibi, Swee-Hua Erin Lim, Kok-Song Lai

**Affiliations:** 1 Department of Cell and Molecular Biology, Faculty of Biotechnology and Biomolecular Sciences, Universiti Putra Malaysia, 43400 Serdang, Selangor, Malaysia; 2 Department of Microbiology, Faculty of Biotechnology and Biomolecular Sciences, Universiti Putra Malaysia, 43400 Serdang, Selangor, Malaysia; 3 Department of Aquaculture, Faculty of Agriculture, Universiti Putra Malaysia, 43400 Serdang, Selangor, Malaysia; 4 School of Pharmacy, Monash University Malaysia, Jalan Lagoon Selatan, Bandar Sunway 47500, Selangor, Malaysia; 5 Health Sciences Division, Abu Dhabi Women’s College, Higher Colleges of Technology, 41012 Abu Dhabi, UAE

**Keywords:** terpenes, terpenoids, antimicrobial resistance, synergy

## Abstract

The evolution of antimicrobial resistance (AMR) in pathogens has prompted extensive research to find alternative therapeutics. Plants rich with natural secondary metabolites are one of the go-to reservoirs for discovery of potential resources to alleviate this problem. Terpenes and their derivatives comprising of hydrocarbons, are usually found in essential oils (EOs). They have been reported to have potent antimicrobial activity, exhibiting bacteriostatic and bactericidal effects against tested pathogens. This brief review discusses the activity of terpenes and derivatives against pathogenic bacteria, describing the potential of the activity against AMR followed by the possible mechanism exerted by each terpene class. Finally, ongoing research and possible improvisation to the usage of terpenes and terpenoids in therapeutic practice against AMR are discussed.

## 1. Introduction

The increase of antimicrobial resistance (AMR) in microbiological pathogens has spurred a global mandate to identify potentially effective alternatives [1]. AMR is defined as inefficacious infection-associated treatment with an antimicrobial agent that used to be effective [2]. The rise of AMR is contributed by both intrinsic and extrinsic factors. For instance, evolution of intrinsic factors in microbes include development of structural attributes [3] such as microbial biofilm production [4], and insertion of transposons [5]. On the other hand, extrinsic contributing factors include excessive antibiotic usage resulting from non-judicious prescribing practices, fueled by increased competition in the production and marketing of antimicrobials within the pharmaceutical industry [6]. As a whole there is also inadequate public education, in tandem with a lack of consistent regulatory systems in place. Both of these, coupled with improper infection control in healthcare, poor sanitation, and water hygiene in low-middle income countries (LMIC), are expanding the AMR challenge [1,7].

Laxminarayan et al. [7] reported that the antibiotic usage in growth and disease prevention on veterinary, agriculture, aquaculture, and horticulture are the main contributors in the non-clinical setting. In the clinical settings however, lack of antibiotic stewardship and uncertain diagnoses by physicians add to emerging pathogen resistance. In fact, as far back as in 1959, the potentially adverse consequences related to antibiotic misuse resulting in selection pressure to the development of resistance were observed. Recent genetic mutations in pathogens which were aided by chromosomal genes and inter species gene transmission has resulted in the rise of resistant microbes such as methicillin resistant *Staphylococcus aureus* (MRSA), *Escherichia coli* ST131 and *Klebsiella* ST258; this further contributes to the dissemination of resistant genes such as *Klebsiella pneumoniae* carbapenemase (KPC), NDM-1, and Enterobacteriaceae-producing extended-spectrum β-lactamases (ESBL) [7].

The continuous dissemination of AMR not only contributes to new resistance mechanisms; it will also have a detrimental impact whereby the efficacy of current antibiotics are drastically reduced, leading to therapeutic failure [8]. It is worrisome to note that in 2010, there were almost 1000 resistant cases worldwide associated with β-lactamases, a 10-fold increase since 1990 [9]. Correlation between antibiotic misuse and AMR is clearly evidenced when quinolone misuse caused the revival of MRSA 30 years after it was first introduced in 1962, while carbapenem misconduct via overuse causing resistance in Enterobacteriaceae has significantly increased over the past decade [7]. In addition, loss of function of ampicillin and gentamicin under the World Health Organisation (WHO) recommended dosage in neonatal infection-related pathogens such as *Klebsiella* spp. and *E. coli* was common in the hospitals of developing countries [10]. This was attributed to the high mortality rates of sepsis cases caused by carbapenem-resistant *Enterobacteriaceae* and *Acinetobacter* spp. in neonatal nurseries. It was also noted by Saleem [11] that in Pakistan, common oral antibiotics such as cefixime and ciprofloxacin have become inefficient in Gram-negative pathogens such as *E. coli*, bacteria commonly associated with urinary infection. Barbieri [2] stated in his review that dissemination of AMR inadvertently affected health systems in the community in LMICs due to the escalating cost of accessing necessary therapies and the prolonged duration of illness caused by AMR which increases treatment time.

**Figure 1:**
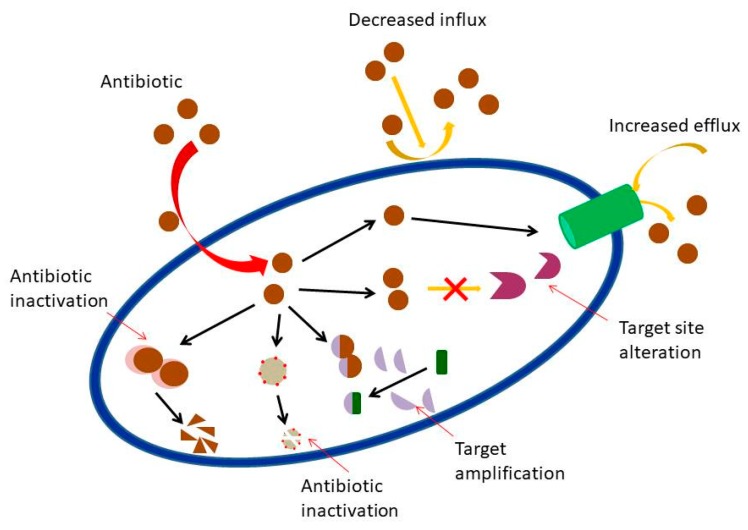
Overall mechanisms of antibiotic resistance in bacteria. Picture adapted from [17].

Bacterial resistance is commonly mediated by transfer of resistance genes [12]. Overall, there are four main ways in which resistance is acquired, firstly, via inactivation of the drug (Figure 1) as reported by Shen [13]. The modification of the antibiotics which occurred based on their target location (bacterial cell wall, cytoplasm, and genome) rendered ineffective to that antibiotic [14]. The second method is the specific modification (Figure 1) at the target such as penicillin-binding protein (PBPs) in MRSA [15]. Similar modifications may arise from mutational or post-translational modifications [3]. Furthermore, porin mutation causes reduction in the number of porins, preventing antibiotic entry and thus increased resistance to antibiotics [16]. Mutations can either be acquired from existing genes (vertical transfer) or new genes can be acquired from other cells (horizontal gene transfer) [17]. Third is the ability of the bacteria to obtain genes for metabolic pathways, these genes then prevent antimicrobial agents from binding to their target. For instance, mobile genes in resistant *Enterococcus* spp. can be disseminated to susceptible strains via horizontal transfer mediated by conjugative plasmids [18]. Finally, the fourth method of bacterial resistance is the reduction of antimicrobial agent intracellularly due to the presence of a bacterial efflux pump. In fact, some resistant bacteria increase impermeability in the cell membrane or increase active efflux, both of which result in reduced drug concentration (Figure 1) in the bacterial cell [19]. The up-regulation in expression of efflux pump has been found to be a major resistance mechanism in many bacteria [20].

Strategies to curb the ongoing emergence of AMR require the involvement from various parties. They include policy makers to develop antibiotic regulations, pharmacists and physicians to practice proper antibiotic stewardship, the pharmaceutical industry to invest in new antibiotic discovery, as well as academics to provide adequate public education [7]. Some strategies for control and containment of AMR included extensive surveillance of antimicrobials, especially for antibiotic prescriptions amongst health care providers, avoiding unnecessary use of antimicrobials in the agricultural sectors, limiting drugs advertising, improvising on sanitation, in tandem with continuous research of novel drugs and invention of nanotechnology; these were some of the measures to be implemented [7]. A shift in focus towards alternative therapies targeting AMR mechanisms would also be an important aspect and these include incorporation of antimicrobial peptides (AMPs), phage therapy, metalloantibiotics, lipopolysaccharides, efflux pump inhibitors, and phytochemicals [1].

Antimicrobial agents may comprise of naturally-occurring compounds such as phytochemicals and essential oils (EOs) [21]. They can also be either semi-synthetic or synthetic [22] in nature. Natural secondary metabolites which have a molecular weight ≤500 g/mol may have the ability to act as adjuvants for antimicrobials and exhibit synergy effects [2,23]. Exploration of new antimicrobial agents via biotransformation such as through microbial modification may present an important alternative [24]. Combination therapy of an antimicrobial agent with a low molecular weight natural product, such as terpene derivatives have shown promising effects, with the ability to eliminate fungal and bacterial biofilm production [4]. Terpenes and their derivatives are secondary metabolites which are commonly found in EOs and have been shown to have antimicrobial activities against susceptible and resistant pathogens [25]. Combination therapy between natural compounds and drugs may be able to recover the loss of function for existing antimicrobial agents [26], potentiating the action of drugs. Wagner and Merzenich [27] reported that the potentiation of antimicrobial agents was accomplished via several mechanisms in combinatorial therapy; these provide a multi-targeted pharmacokinetic effect, allowing simultaneous destruction of existing resistance mechanisms in a specific pathogen.

EOs are naturally produced from aromatic plants such as herbs as their secondary metabolites. Usually EOs exist in liquid form, are volatile and exhibit good solubility in lipids and inorganic compounds that are less dense compared to water. They can be extracted from various parts of the plant organ such as flowers, buds, leave, bark, twigs, stem, wood, seed, or root [28] by various methods which include solvent extraction (solvent, supercritical CO_2_, subcritical water), distillation (steam, hydrodistillation, hydrodiffusion), solvent-free (microwave) and combination method (solvent + steam) [29]. Generally, plants store their EOs in specific cellular compartments such as in the secretory cells, cavities, or glandular trichomes. EOs primarily function as protection against plant pests and infections [30]. In particular, EOs have been reported to be a prominent antimicrobial, antioxidant, and insecticidal agent, significantly inhibiting microbial biofilm production and the growth of bacteria, yeasts, and molds [31]. Previously our group has also focused on the bacterial membranous disruption effect when subjected to treatment with EOs [32]. A list of reports summarized in Table 1 indicate various antimicrobial activities possessed by EOs. In 2018, our research group demonstrated synergistic activity when peppermint (*Mentha x piperita* L. Carl) essential oil was added to meropenem against resistant *E. coli* [33]. Recently, our research group established a mode of action of EO from cinnamon bark against KPC-KP via oxidative stress [34]. There are challenges involved in working with EOs. They are laborious to handle as they need to be extracted and purified before being tested and manipulated. Furthermore, despite EOs being used for testing, it is difficult to ascertain as to which bioactive component in EOs is contributing to the antimicrobial activity. Acquisition of EOs will also require higher cost as compared to synthetic additives because they need to be processed prior to screening for their activity. One method to resolve the cost issue involves downscaling the volume via extraction of the antimicrobial compounds. This review in particular will focus on one such compound commonly found in EOs, namely the terpenes and their antimicrobial potential and their possible mechanisms of action.

## 2. Terpenes and Their Derivatives

Terpenes are large hydrocarbon groups that consist of 5-carbon isoprene (C5H8) units as their basic building block. They are synthesized via two pathways which are the non-mevalonate pathway; the Methylerythritol Phosphate (MEP) and the mevalonate pathway from Acetyl CoA precursor. Their backbones can be reorganized into cyclic structure by cyclases. The commonly found terpenes which differ in numbers of isoprene units are the monoterpenes and sesquiterpenes; however longer chains such as diterpenes and triterpenes also exist [47,54]. P-Cymene, limonene, sabinene, terpinene, carene, and pinene are examples belonging to the terpene groups. Most terpenes possess reduced antimicrobial activities [54]. Terpenoids are derivatives of terpenes which takes place when modification of terpenes occur, such as with the addition/removal of functional groups [2]. Therefore, the antimicrobial activity of terpenoids are determined from their functional group [55]. For instance, the shifting or removal of a methyl group and addition of oxygen by a specific enzyme result in derivation of terpenes. The hydroxyl group of the phenolic terpenoids and delocalized electrons are amongst the antimicrobial determining factors. Linalool, menthol, carvacrol, thymol, linalyl acetrate, piperitone, geraniol, and citronella are amongst the best studied terpenoids.

### 2.1. Bioactive Terpenes and Terpenoids

Terpenoids represent a large group of phytochemicals with promising antimicrobial activity [2]. The chemical diversity of terpenoids have led to discovery of over 40,000 structural varieties, with a few classes serving as pharmaceutical agents, some of which include terpenoid derived indole alkaloids [56]. There are a total of eight different classes of terpenoids (hemiterpenoids, monoterpenoids, sesquiterpenoids, diterpenoids, sesterpenoids, triterpenoids, tetrapenoids, and polyterpenoids) which differ in the number of isoprene (C5H8) units. Recently in 2017, it was reported that 67% of potentiators belong to monoterpenes and sesquiterpenes [4]. Meanwhile, specifically among the discovered potentiators of antibacterial drugs, 75% were terpenes; these include classes of mono-, di-, and tri-terpenes [4].

Although the antibacterial mode of action of terpenes remains largely unknown, Griffin et al. [55] reported in his study that most terpenoids are able to inhibit two crucial processes which are essential to microbial survival, this includes oxygen uptake and oxidative phosphorylation. Aerobic microbes require oxygen in order to yield energy for their growth. Previously, it was proven that low oxygen concentrations caused limitation in bacterial respiration rates [57]. Meanwhile, oxidative phosphorylation is a crucial biochemical process responsible for cellular respiration that takes place in the cytoplasmic membrane. Thus, terpene interaction leads to alteration in cellular respiration which later causes uncoupling of oxidative phosphorylation in the microbe [58]. Additionally, carbonylation of terpenoids was believed to increase bacteriostatic activity but not necessarily the bactericidal activity. A bacteriostatic agent is an agent that stops or inhibits microbial growth, while a bactericidal is responsible for killing the microbe. Terpenoids have also been found to exhibit antiseptic potential according to their solubility in water. Lipophilicity and/or hydrophobicity and presence of hydroxyl groups in the terpenes are amongst the determining elements of their antibacterial action [58]. In skin barrier-associated treatment, terpenes have also been reported to affect the lipid membrane activity by interacting with lipophilic tails of intermembrane lipid and polar head groups which, at the end, affects the lipodial intermembrane and polar transmembrane pathways [59].

#### 2.1.1. Monoterpenes and Monoterpenoids

Monoterpenes comprise of two isoprene units and exist in many plants. It has been reported by Griffin et al. [55] that monotepenes possess antimicrobial activity. For instance, carvacrol, thymol, menthol, and geraniol were able to work against Gram-positive and Gram-negative bacteria. Geraniol was also later claimed to efficiently increase the susceptibility of the Gram-negative multi-drug resistant (MDR) *Enterobacter aerogenes* by becoming a potent efflux pump inhibitor [60]. Trombetta et al. [61] claimed in his study that three monoterpenes linalyl acetate, (+) menthol and thymol showed positive responses against *S. aureus* and *E. coli.* Other compounds such as carvacrol, trans-cinnamaldehyde and (+)-carvone were reported to possess potent inhibitory activity against *E. coli* and *S. typhimurium* [30]. In addition, other types of monoterpenes such as halogenated monoterpenes recorded good cytotoxic, antimalarial, and antialgal effects while monocyclic monoterpenes had been reported to exhibit potent insecticidal, as well as antifungal effects [62].

In fact, in as early as 1979, Kurita et al. [63] had listed a total of 13 monoterpenes ((+)-terpinen-4-ol, γ-terpinene, α-terpinene, terpinolene, α-pinene 1,8-cineole, ρ-cymene, (+)-limonene, β-myrcene, (+)-β-pinene, (±)-linalool, α-phellandrene, α-terpinoel) which exhibited antifungal properties against 14 fungal strains. The phenolic monoterpenes such as carvacrol, eugenol and thymol were found to be highly active against bacteria [64]. There were twenty one monoterpenes (borneol, d-3-carene, carvacrol, carvacrol methyl ester, *cis/trans* citral, eugenol, geraniol, Geranyl acetate, *cis*-hex-3-en-1-ol, R(+)limonene, (2)-linalool, menthone, nerol, α-pinene, β-pinene, (+)sabinene, α-terpinene, terpinen-4-ol, α-terpineole, (−)-thujone, thymol) which were previously reported regarding their antimicrobial activity against 25 bacterial strains [65]. Phenol monoterpenes, such as carvacrol were also reported to inhibit biofilm development of *S.aureus* and *S. typhimurium* [66]. Recently, our research group found one monoterpene compound, linalool, extracted from lavender essential oil which exhibited strong antimicrobial activity against resistant *K. pneumoniae* [34]. The proposed mechanism of action for the compound was membrane disruption [34].

Monoterpene ketones were also found to exhibit antimicrobial properties [67]. In comparison, alcoholic monoterpenes are more bactericidal agents rather than bacteriostatic agents. In line with this, Bhatti et al. [67] reported that monoterpenes alcohol of terpinen-4-ol, α-terpineol, 1, 8-cineole and linalool exhibited good antifungal activity and suggested alcohol moieties as determinants of antifungal activity. In addition, myrcene, one of the acyclic monoterpene alcohols showed a negative response against fungal specimens; this infers that the cyclic structure of monoterpenes may also be the structure responsible for this activity [68]. Monoterpenes consisting of aldehydes, however, possessed a potent antimicrobial activity which can be explained through its carbon double bond arrangements; this creates high electronegativity. The observations of nine monoterpenes (α-terpinene, γ-terpinene, α-pinene, ρ-cymene, terpinen-4-ol, α-terpineol, thymol, citral and 1, 8-cineole) against *Herpes simplex virus* type 1 (HSV-1) were made by Thompson in 1989. Later in 2010, Dunkic et al. [69] listed a few more monoterpenes having considerable activity against HSV-1 which are borneol, bornyl acetate, and isoborneol, 1, 8-cineole, thujone, and camphor. Thymol and carvacrol were also noted to be powerful agents against the *Tobacco mosaic virus* (TMV) and *Cucumber mosaic virus* (CMV) [70].

#### 2.1.2. Sesquiterpenes and Sesquiterpenoids

It has been long recognized that sesquiterpenes possess antimicrobial activites [55]. Back in 2011, Torres-Romero et al. [71] identified one of dihydro-β agarofuran sesquiterpenes, namely 1α-acetoxy-6β, 9β-dibenzoyloxy-dihydro-β-agarofuran inhibits the growth of *Bacillus* spp. [71]. Farnesol, which is an isoprenoid natural acyclic sesquiterpene alcohol showed moderate effects against *Streptococcus mutans* and *Streptococcus sobrinus* biofilm formation [72]. Farnesol also showed antibacterial activity against *S. aureus* and *S. epidermidis* whereby it inhibited the biofilm development [73]. Two studies conducted by Masako [74] evidenced that combinations of farnesol with xylitol have positive effects against atopic dermatitis caused by *S. aureus* without altering the microbial flora and successfully inhibited the biofilm production of *S. aureus*. Sesquiterpenes were also incorporated in the combination therapy using existing drugs.

A recent study conducted by Castelo-Branco et al. [75] showed potentiation effects of a combination therapy of farnesol with amoxicillin, doxycycline, ceftazidime, and sulfamethoxazole-trimethoprim against *B. pseudomallei*. A phenol sesquiterpene, xanthorrhizol was found to reduce 60% of *Staphylococcus mutans* cell adherence ability [76], and inhibited the growth of *Mycobacterium smegmatis* [77]. Recently, it was discovered that sesquiterpenes have potent antibiotic enhancement against MRSA and also Gram-negative bacteria [4]. In 2011, Gonçalves et al. [78] reported a significantly larger inhibition zone when sesquiterpenes were incorporated into antibiotic discs. The experiment was conducted against MDR strains of *S. aureus* with a combination of sets of available antibiotics such as tetracycline, erythromycin, penicillin, and vancomycin.

#### 2.1.3. Diterpenes and Diterpenoids

Diterpene is a class of terpene with broad biological activities [79]. Previously, 60 terpenoids have been tested for their minimum inhibitory concentration (MIC) against *P. aeruginosa, E. coli, S. aureus,* and *C. albicans* [55]. They were then classified into five groups to determine their activity patterns. Hydrogen bond was found to be the factor that determines the positive antimicrobial activity. On the other hand, low water solubility was discovered to be the factor of antimicrobial inactivity. Griffin [55] suggested that inhibition of microbial oxygen uptake and oxidative phosphorylation are likely mechanisms of action responsible for the antimicrobial properties of the diterpene class. Separately, diterpene derivatives such as ent-kaurane and ent-pimarane are able to inhibit growth of the dental caries pathogens. The MIC value of 2–10 mg/mL confirmed the antibacterial potential of the compounds [80]. Additionally, the diterpenoid salvipisone prevented cell adherence and biofilm developments of *S. aureus* and *S. epidermidis* [81].

Besides sesquiterpenes, diterpenes also function as a good antibiotic enhancer against MRSA. Moreover, diterpenes have been widely used in combination therapy with antibiotics [82]. For instance, clerodane diterpenoid 16αhydroxycleroda-3, 13 (14)-Z-dien-15, 16-olide (CD) extracted from leaves of *Polyathia longifolia* enhanced the efficacy of oxacillin, tetracycline, daptomycin, and linezolid against clinical isolates of MRSA. All MICs of the antibiotic dropped significantly between 10–80, 4–16, 2–8 and 2–4-folds respectively when combined with CD. Gupta et al. [82] then proposed the in vivo mechanism of CD in reversing the resistance of clinical isolates of MRSA. The same clinical MRSA isolates were tested with CD combined with norfloxacin, ciprofloxacin, and ofloxacin. qRT-PCR analysis showed that the expression of genes coding for efflux pumps were significantly modulated in cells treated with CD alone and in combination with antimicrobial drugs. In fact, the results of time-kill assay showed the MIC in combination of CD with norfloxacin was half of the MIC of CD and norfloxacin alone, undoubtedly decreasing the viability of bacterial cells. Unfortunately, despite the promising effects offered by CD, sourcing to obtain CD became the bottleneck for further testing [4].

Salvipisone and aethiopinone are diterpenoids isolated from roots of *Salvia sclarea* [81]. They were shown to express antibacterial and antibiofilm activities against *S. aureus, Enterococcus faecalis* and *S. epidermidis.* Both salvipisone and aethiopinone were also tested for their synergistic activity when combined with antimicrobial drugs alongside oxacillin, vancomycin, and linezolid against MRSA and Methicillin resistant *Staphylococcus epidermidis* (MRSE). It was discovered that they were either bactericidal or bacteriostatic against planktonic cultures of tested MRSA and MRSE [83]. Remarkably, the MIC was achieved with 50% reduction in the dose of antibiotic when diterpenoids were used in combination.

#### 2.1.4. Triterpenes and Triterpenoids

Triterpenes comprises of six isoprene units. It was reported that *Pandanaceae* containing triterpenes in the form of 24, 24-dimethyl-5β-tirucall-9 [11], 25-dien-3-one showed promising activities against tubercular strains. Another triterpene, Oleanic acid (OA) is potent against pathogens such as *Mycobacterium tuberculosis.* OA also had promising synergy against MDR when combined with rifampicin, isoniazide, and ethambutol with significant MIC reduction of 128–16 fold, 32–4 fold, and from 128 to 16 fold, respectively [2].

Besides OA, bonianic acid A and B are two triterpenoids that were extracted from *Radermachera boniana.* Both compounds were found to be active against *M. tuberculosis*. There are at least six known molecules which include OA, ergosterol peroxide, and ursolic acid (UA). In fact, the combination of both ergosterol peroxide and UA showed synergistic activity against *M. tuberculosis* [84]. The reports conducted by Cunha et al. [85] depicted that OA and UA that were isolated from *Miconia ligustroides*, resulted in significant antibacterial activity when tested on selected bacteria (*B. cereus, Vibrio cholerae, S. choleraesuis, K. pneumoniae and S. pneumoniae*). When UA was used against *B. cereus,* the MIC value was 20 µg/mL and OA showed MIC value of 80 µg/mL against *B. cereus* and *S. pneumoniae*. In 2013, a study conducted by Zhou et al. [86] showed that UA and OA were active against planktonic cariogenic microorganism and their biofilm. Later in 2015, Liu et al. [31] expanded the research and reported the combinatory effects of UA and xylitol against biofilm produced by *S. mutans and S. sobrinus*. Moreover, OA and UA were reported to enhance antimicrobial activity against *Listeria monocytogenes* without affecting toxin secretion; this influenced the virulence factors of *L. monocytogenes* and inhibited the capacity of biofilm production from these bacteria [45].

OA also exhibited strong interactions alongside aminoglycoside (gentamicin and kanamycin) against *A. baumanii*, but not with other classes of which ampicillin, norfloxacin, chloramphenicol, tetracycline, and rifampicin are examples [4]. Based on time-kill assay, the bactericidal effects of gentamicin were significantly greater when combined with OA compared to gentamicin alone [4]. Three triterpenoids, amyrin, betulinic acid, and betulinaldehyde were extracted from the bark of *Callicarpa farinose* Roxb (Verbenaceae) and were shown to exhibit potent antimicrobial activity against clinical methicillin-resistant (MRSA) and methicillin-susceptible (MSSA) with MICs ranging from 2 to 512 µg/mL [87].

**Figure 2:**
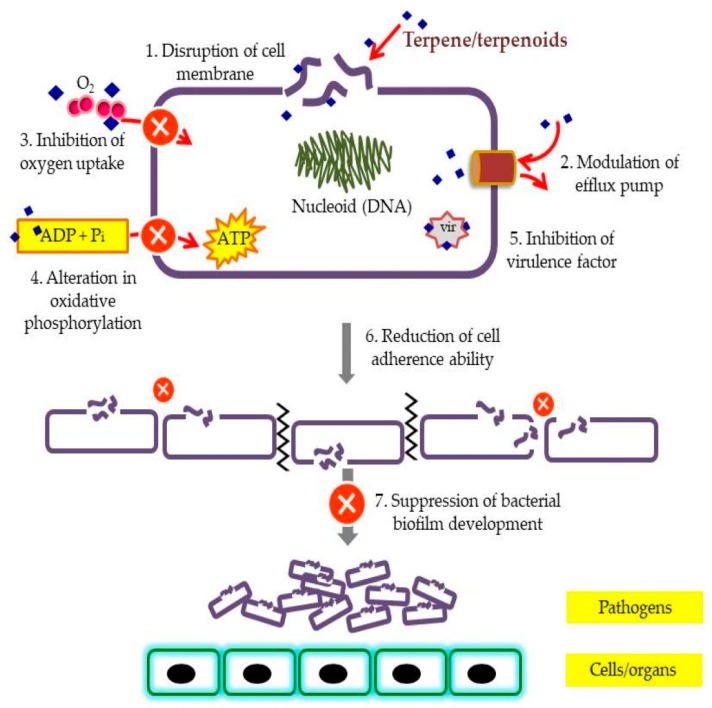
Postulated mode of action of terpene/terpenoids on antibiotic resistance pathogens and as combination therapies.

While there is no firm report specifically on modes of action by terpenoids, the mechanisms of action of phytochemicals found in nature have been proposed. Typically, phytochemicals aim either for disruption of the bacterial cell membranes, modulation of bacterial efflux pump, suppression of bacterial biofilm development or inhibition of some virulence factors which include enzymes and toxins [2]. For instance, carvacrol was found to be responsible for sub-lethal injury to bacterial cells due to alteration of fatty acid compositions, while other reports state that carvacrol and thymol caused disintegration of the outer membrane and disruption of the cytoplasmic membrane of Gram-negative bacteria [30]. Antimicrobial activity effects of some terpenoids are summarized in Table 2 while the postulated mode of action of terpenes on antibiotic resistance pathogens and as combination therapies are depicted in Figure 2.

### 2.2. Therapeutic Implementation

#### 2.2.1. Drugs and Antibiotics

Combination therapy with terpenes have been widely seen in current therapeutic practice especially in antifungal drugs [26]. It was shown that fluconazole which had once lost its efficacy, had been potentiated by monoterpenes, thymol, and carvacrol when subjected against 38 fluconazole-sensitive *C. albicans, C. tropicalis*, and *C. glabrata* and 11 fluconazole-resistant *C. albicans, C. krusei, C. glabrata, C. tropicalis,* and *C. parapsilopsis*. The combination analysis showed that of the strains tested, 32 out of 38 strains and eight out of 10 strains have obtained a Fractional Inhibitory Concentration (FIC) index of less than 0.5 [88]. FIC is a term used to express the degree of synergy interaction between antibacterial drugs whereby FIC < 0.5 shows positive synergism while FIC > 0.5 shows negative synergism [89]. The sequiterpene, farnesol, also showed potentiation activity with fluconazole against candidiasis [88]. Previously, it was reported that three diterpene compounds, ent-clerodanes (bacchotricuneatin, bacrispine and hawtriwaic acid) which were isolated from *Baccharis* extract synergistically reduced the dose of the anti-fungal drug Terbinafine against *Trichophyton rubrum* [90]. Another triterpene, retigeric acid, found in the lichenized fungi family, Lobariaceae exhibited strong potentiation when combined with either fluconazole, itraconazole or ketoconazole against azole-resistant *C. albicans* strains [91]. It was proposed that facilitation of azole uptake or membranous repair associated with azoles were the modes of action of retigeric acid.

Prior to the development of novel drugs, in vitro and in vivo testing are usually performed to ascertain the safety and efficacy of the compound to better understand the physiological effects. Despite a number of published in vitro reports pertaining to terpenes antimicrobial testing, incorporation of various terpenes in clinical trials focusing on antimicrobial activity is still lacking due to insufficient data on the in vivo system. Most in vivo testing for terpenes and its derivatives has been conducted for human health associated with anti-inflammatory, anti-tumorigenic, anti-cancer, transdermal delivery medium and neuroprotective [92] aspects. However, incorporation of terpenes into household products and cosmetics due to antibacterial properties showed increasing assurance in vivo, inhibiting multiple species of bacteria [93]. In 2006, Mondello et al. [94] demonstrated in vivo activity of the monoterpene terpinen-4-ol which is the main bioactive constituent of *Malaleuca alternifolia* Cheel (tea tree) oil against azole-susceptible and resistant human pathogenic candida species. In this demonstration, terpinen-4-ol was able to clear a well-established model of rat vaginal candidiasis. Terpenes which were found in *Cassia occidentalis* and *Phyllanthus niruri* showed antimalarial activity in vivo using mice against *Plasmodium berghei* [95]. In addition, β-sitosterol was tested in the treatment of culture proven pulmonary tuberculosis (PTB) patients using blinded randomized placebo-controlled trials. Two groups of patients consisting of a sitosterol group and a placebo group were set up upon the treatment. Patients were hospitalized for the duration of treatment and checked monthly with regards to sputum culture positivity, chest radiography, weight gain, hematology, liver function, and Mantoux test response. At the end of trials, it was reported that the sitosterol group marked a greater weight gain, lymphocyte and eosinophils count compared to the other group [96].

#### 2.2.2. Terpenes Bioavailability

In order to ensure greater therapeutic effect from drugs, terpene bioavailability should be determined. It was reported that while natural volatile terpenes from 1,8-cineole of uncrushed capsule from the plasma yielded relatively 100% of bioavailability, limomene and α-pinene were only detectable for a few subjects [97]. In 2017, research conducted by Papada et al. [98] demonstrated positive bioavailability of major terpenes from Mastiha powder after 30 min of ingestion with the highest peak between 2–4 h post ingestion. The plasma analysis was done using ultra-high-pressure liquid chromatography high-resolution MS (UHPLC-HRMS/MS). The bioavailability bottleneck of medicinal herbs including terpenes, however, had been improved ever since phytosome technology arrival. It was reported that the bioavailability of *Ginkgo biloba* extract (GBE) which constitutes of the terpene, lactone, was improved significantly with 2–4 times greater plasma concentration compared to a non-phytosome delivery method [99].

#### 2.2.3. Evaluation of Compounds Interaction in Combination Therapies

Combination therapies using natural products such as the terpenoids may synergistically, additively, or antagonistically affect the treatments. Zacchino et al. [4] reported in a review that the nature of interaction between phytochemical and antimicrobial drugs can be determined using the median-effect method of Chou [89] which permits the calculation of combination index (CI). As for combination therapy, both agents at a fixed ratio will result in IC_50_ respectively; these are mixed with two-fold dilutions of both agents with a fixed ratio. The CI will resolve synergistic (CI < 1), additive (CI = 1) and antagonistic effects (CI > 1). In addition, another method that contributed significantly in synergistic activity was to calculate the Dose Reduction Index (DRI), also known as the reversal enhancement ratio, that measures how many folds the dose of antimicrobial drugs may be cut down when used in combination rather than alone. One of the measures that can be used to evaluate synergism is through checkerboard assay.

In our previous study, through this method, peppermint essential oil was proven to synergistically react with meropenem. MIC of individual peppermint oil and meropenem were 8% and 4 µg/mL respectively. Meanwhile when used in combination, the MIC of peppermint oil and meropenem were reduced to 1% and 0.5 µg/mL respectively. The CI value of 0.26 obtained in checkerboard assay had portrayed a high synergism. Last year, our group carried out extensive analysis to investigate the additive interaction of cinnamon bark oil and meropenem. The shift of attention towards synergism between compounds and antibiotics have caused researchers to overlook the additive effects, thus we conducted the study to understand additive interaction which focused on the effect on the bacterial membrane [100].

#### 2.2.4. Methods for Antimicrobial Evaluation

Both in vitro and in vivo experimental systems can be used to evaluate the antimicrobial activity of either synthetic compounds or naturally-acquired compounds. Nevertheless, in vitro approaches have been more commonly used due to their feasibility. In vivo studies, however, are seldom applied due to limitations in detecting the actual mechanism of action. Susceptibility testing which determines the MIC of a compound against bacteria is routinely done using variations in the methods of MIC assay such as rapid *p*-Iodonitrotetrazolium chloride (INT) colorimetric assay, micro- or macro-dilution and disc diffusion methods. However, Griffin et al. [55] stated in his study that the disc diffusion method is prone to problems as the method was highly dependent on water solubility and suitability of the test agent to be diffused through the agar. In combination therapy, however, the effects are assessed through the checkerboard assay which investigated the interaction between agents. The checkerboard antibiofilm microsomal triglyceride transfer protein (MTP) assay through which the checkerboard microdilution was seeded with biofilm have also been used in an experiment associated with biofilm producing bacteria [88]. By performing the checkerboard assay, an important index called the FIC will reveal the potential of an individual compound [4]. The Dose Reduction Index (DRI) [89] can also be conducted in a compound combination analysis in order to find out the dosage reductions ruled out by individual compounds that affected the MIC of the second compound. A greater DRI disclosed better adjuvant capabilities for a given effect level [89]. More extensively, further analysis usually includes isobolograms and time-kill studies.

## 3. Perspectives

### 3.1. Ongoing Research

Effective management and treatment of microbial resistance are among the main priorities in healthcare. Terpene derivatives are an important and promising source of novel antibiotics. Indeed, ent-kaurenoids (ent-kaur-15-en-18, 20-diol and ent-kaur-15-en-18-ol) extracted from *Senegalia nigrescens* are among the novel terpene derivatives discovered recently. Both in vitro and in silico anti-quorum sensing evaluation have demonstrated potential anti-quorum sensing against *Chromobacterium violaceum* [101]. In addition, it was reported that antiquorum sensing does not contribute towards evolution of MDR pathogens as there is no enforcement of selection pressure [32]. Additionally, terpenoids found in microbial volatile compounds (MVCs) have exhibited the ability to combat and modulate antibiotic resistance in human and animal pathogens [102].

With the success of terpenoids in the treatment of microbial resistance, the hunt for new terpenoids has been an important quest amongst the scientific community as a potential application. For instance, screening for terpenoids was conducted on semi-arid plants such as *Caesalpinia pulcherrima, Lawsonia inermis, Pithecellobium dulce, Euphorbia tithymaloides, Punica granatum, Plumeria obtusa, Carica papaya, Cassia fistula, Cordia dichotoma, Euphorbia prostrate, Nerium oleander*, and *Cyanthillium cinereum* [103]. A separate study conducted in 2018 identified three terpenoid derivatives (α-pinene—45.44%, 3-carene—38.34%, and terpinolene—5.36%) of *Cupressus torulosa* essential oil. The compounds are effective against pathogens including *B.subtilis, Pseudomonas alcaligenes, M. luteus,* and *B. cereus* [104]. This can mitigate AMR problems by manipulating combinatory therapeutics of existing antimicrobial agents with terpenoid derivatives. *Ganoderma lucidum* (Reishi) which is a medicinal mushroom, contains several triterpenoid substances such as ganoderic acid and lucidenic acid. The compounds were then evaluated for their therapeutic effects whereby they exhibited anti-human immunodeficiency virus (HIV) activity by inhibiting the effects of HIV progression [105].

### 3.2. Application of Terpenoids in Clinical Settings: Challenges

As mentioned previously, terpenes and terpenoids had been known to exert antimicrobial activity against a wide variety of bacteria, both Gram-positive and Gram-negative. Clinical trials regarding highlighting the application of terpenes had been performed in several studies. In addition, β-sitosterol had also demonstrated immune enhancing ability in tuberculosis patients, demonstrating significant weight gain and higher white blood cell counts which resulted in faster recovery [106]. However, the application of terpenes as antimicrobials in the clinical phase is yet to be explored. This can be attributed to several factors, as the mode of actions of terpenes is not fully understood and the amount of time and resources required for clinical trials are limited and not always rewarding [107]. Terpenes consist of a diverse group of lipophilic organic compounds, resulting in different structures which affect their mode of action. β-caryophyllene showed poor antimicrobial activity against a panel of bacteria [108]. In the event whereby terpenes with efficient antimicrobial activities have been discovered, the safety of the terpenes would often be the next obstacle prior to clinical trials. Certain terpenes are reported to be toxic at low dosage and thus not preferred [109]. For instance, even at 1% dose of eugenol, it was reported to effectively inhibit growth of *Dermanyssus gallinae* at 20% of the pathogen population. Eugenol, geraniol, and citral found in plant essential oils were able to administer 100% mortality when used undiluted. This shows that some undiluted terpenes are highly toxic upon direct usage [110]. Generally, the unfavorable toxicity of terpenes towards whole cells takes place due to disturbance; primarily disrupting cell membrane integrity which eventually leads to cell lysis [111]. Due to the lipophilic nature of terpenes, upon ingestion, they are easily absorbed by epidermal cells before reaching the site of infection. Thus, delicate drug delivery systems are required for their application into clinical trials.

### 3.3. Future Prospects

The evidenced antimicrobial activity of terpenes and their derivatives need to be further expounded with the aid of automation and advancement in technology. Experimental analyses will need to be more streamlined to become more precise, resulting in less ambiguity so that the results obtained can be ensured and are consistent. This will reduce the time taken for experimental work and more time can be spent for extended analyses. Researchers may then study the modification effects of natural products with a special focus on terpenoids. Structural modifications of natural compounds produced either synthetically or via biotransformation may offer a new facet in finding novel AMR solutions as it explores new antimicrobial agents. In addition, delivery methods involving existing treatments against AMR should be improved and new inventions can be investigated. Combination therapeutics may be enhanced by exploring more antimicrobial adjuvants which will synergistically affect treatment outcomes with greater efficiency and less side effects. Natural terpenes and terpenoids which are available at very low prices such as carvacrol, thymol, and geraniol [4], should be optimally used for development of good antibacterial combination drugs. However, application at the pharmaceutical level remains challenging as the in vivo after effect is, currently, still very much unexplored. Extended analysis involving well-designed clinical trials should be improved in order to manipulate potent compounds of terpenoids to the best of their functional potential.

## 4. Conclusions

From this review, it has been evidenced that some terpenes and their derivatives were proven to be potent antimicrobial agents against drug resistant pathogens which mainly include bacteria and fungi. Specific mechanisms of each class of terpenes have also been highlighted and as a whole, terpenes provide a possible mitigation route for AMR and navigating the dead end of the diminishing antibiotic pipeline, hence, an appropriate match between terpenoids and existing antimicrobial agents may provide ultimate therapeutic options for AMR-associated infections.

## Figures and Tables

**Table 1:** Essential oils extracted from plant against tested pathogens.

Plants spp.	Common Name	Pathogens Tested	MIC/Sensitivity/Inhibition Zone	Citation
*Eugenia caryophyllata*	Clove	*Burkholderia cepacia* complex	ES	[35]
*Origanum vulgare*	Oregano	*B. cepacia* complex	ES	[35]
*Thymus vulgaris*	Thyme	*B. cepacia* complex	ES	[35]
*Eucalyptus camadulensis*	Eucalyptus	*Streptococcus pyogenes*	1 mg mL^−1^	[36]
		*Fusarium oxysporum* f. sp. *lycopersici*	15.93% to 72.5%	[37]
*Mentha spicata*	Spearmint	*S. pyogenes*	2 mg mL^−1^	[36]
		*E. coli*	21 mm at 150 µL	[38]
		*Salmonella thyphi*	13 mm at 150 µL	
		*S. aureus*	12 mm at 150 µL	
*Cymbopogon citratus*	Lemongrass	*Acinetobacter baumannii*	0.65% (v/v)	[39]
		*F. oxysporum* f. sp. *lycopersici*	250 ppm	[37]
*Syzygium aromaticum*	Clove	*Candida albicans*	360 µg mL^−1^	[40]
		*F. oxysporum* f. sp. *lycopersici*	125 ppm	[37]
*Pelargonium graveolens*	Geranium	*B. cepacia* complex	0.4% (v/v)	[41]
		*Prototheca zopfii*	3.5 to 4.0 µL mL^−1^	[42]
*Laurus nobilis*	Bay laurel	*S. typhimurium*	3 % (v/v)	[43]
		*E. coli*	1 % (v/v)	[44]
		*Candida* spp.	250 to 500 µg mL^−1^	
*Melaleuca alternifolia*	Tea tree	*Campylobacter* spp.	0.00%	[45]
*Leptospermum petersonii*	Manuka	*Campylobacter* spp.	0.01%	[45]
*Backhousia citriodora*	Lemon myrtle	*Campylobacter* spp.	0.01%	[45]
*Lavandula angustifolia*	Lavender	*S. aureus*	2 mg mL^−1^	[46]
		*Pseudomonas aeruginosa*	2 mg mL^−1^	
		*C. albicans*	3 mg mL^−1^	
*Mentha x piperita*	Peppermint	*Clostridium perfringens Fusarium oxysporum* f. sp. *lycopersici*	10 mg mL^−1^	[47]
			500 ppm	[37]
*Chamaemelum nobile*	Roman chamomile	*Porphyromonas gingivalis*	20.5 ± 0.5 mm	[48]
*Origanum majorana*	Marjoram	*Micrococcus luteus*	0.097 mg mL^−1^	[49]
		*Vibrio alginolyticus*	0.39 mg mL^−1^	
*Foeniculum vulgare*	Fennel	*Candida* spp.	1.56 to 12.48 mg mL^−1^	[50]
*Pinus sylvestris*	Pine	*Pseudomonas* spp.	4.33 ± 0.58 mm	[51]
*Cedrus atlantica*	Cedarwoood	*E. coli*	0.4 µL mL^−1^	[52]
		*Bacillus subtilis*	0.2 µL mL^−1^	
		*Bacillus cereus*	0.4 µL mL^−1^	
*Aniba rosaeodora*	Rosewood	*Trichophyton mentagrophytes*	0.002 M	[53]

ES: extremely sensitive.

**Table 2:** Summary of antimicrobial activity effects of some terpenoid class.

Terpenoids Class	Chemical Compounds	Tested Microorganism	Antimicrobial Effect	Reference
Monoterpenes and monoterpenoids	Carvacrol Thymol Geraniol	Resistant *Enterobacter aerogenes*	Efflux pump inhibition	[60]
	Linalyl acetate (+)- Menthol Thymol	*S. aureus* *E. coli*	Growth inhibition	[61]
	Carvacrol Trans-cinnamaldehyde (+)-Carvone	*E. coli* *S. typhimurium*	Growth inhibition	[30]
	(+)-Terpinen-4-ol γ-Terpinene α-Terpinene Terpinolene α-Pinene 1,8-Cineole ρ-Cymene (+)-Limonene β-Myrcene (+)-β-Pinene (±)-Linalool α-Phellandrene α-Terpinoel	*T. mentagrophytes Trichophyton violaceum Microsporium gypseum Histoplasma capsulatum Blastomyces dermatitidis*…etc.	Growth inhibition	[63]
	Carvacrol Eugenol Thymol	*Acinetobacter calcoacetica* *Aeromonas hydrophila* *B. subtilis*	Growth inhibition	[64]
	Borneol d-3-Carene Carvacrol Carvacrol methyl ester *cis/trans* Citral Eugenol Geraniol Geranyl acetate *cis*-hex-3-en-1-ol R(+)Limonene (2)-Linalool Menthone Nerol α-Pinene β-Pinene (+)sabinene α-Terpinene Terpinen-4-ol α-terpineole (−)-Thujone Thymol	*S. aureus* *E. coli* *Salmonella typhia* *S. typhimurium* *Salmonella enteritidis* *A. hydrophila* *Yersinia* sp. *Vibrio anguillarum* *Shigella* sp. *Vibrio parahaemolyticus C. albicans* *Penicillium expansum* *Aspergillus niger*…etc.	Growth inhibition	[65]
	Carvacrol	*S.aureus* *S. typhimurium*	Biofilm inhibition	[66]
	Linalool	Resistant *K. pneumoniae carbapenemase* (KPC)	Cell membrane disruption	[34]
	Terpinen-4-ol α-Terpineol 1, 8-Cineole Linalool	*A. niger* *Botrytis cinerea*	Growth inhibition	[67]
	α-Terpinene γ-Terpinene α-Pinene ρ-Cymene Terpinen-4-ol α-Terpineol Thymol Citral 1, 8-Cineole Borneol Bornyl acetate Isoborneol 1, 8-Cineole Thujone Camphor	*Herpes simplex virus* type 1 (HSV-1)	Growth inhibition	[69]
	Thymol Carvacrol	*Tobacco mosaic virus* (TMV) *Cucumber mosaic virus* (CMV)	Growth inhibition	[70]
Sesquiterpenes and Sesquiterpenoids	1α-Acetoxy-6β, 9β-dibenzoyloxy-dihydro-β-agarofuran	*Bacillus* spp.	Growth inhibition	[71]
	Farnesol	*Streptococcus mutans Streptococcus sobrinus*	Biofilm formation inhibition	[72]
		*S. aureus* *S. epidermidis*		[73] [74]
		*B. pseudomallei*	Potentiation effect—combination therapy	[75]
	Xanthorrhizol	*Staphylococcus mutans*	Reduction of cell adherence ability	[76]
		*Mycobacterium smegmatis*	Growth inhibition	[77]
Diterpenes and diterpenoids	(-)-Carvone Thymol Dihydrocarveol (-)-Perilla alcohol Carvacrol (-)-Carveol…etc.	*P. aeruginosa* *E. coli* *S. aureus* *C. albicans*	Growth inhibition	[55]
	Ent-kaurane Ent-pimarane	Dental carries pathogens	Growth inhibition	[80]
	Salvipisone	*S. aureus* *S. epidermidis*	Bacterial cell adherence prevention Biofilm development inhibition	[81]
	16αHydroxycleroda-3, 13 (14)-Z-dien-15, 16-olide (CD)	MRSA	Antibiotic potentiation Efflux pump modulation	[82]
	Salvipisone Aethiopinone	*S. aureus Enterococcus faecalis* *S. epidermidis*	Biofilm production inhibition	[81]
		MRSA MRSE	Synergistic activity alongside antibiotic	[83]
Triterpenes and triterpenoids	24, 24-Dimethyl-5β-tirucall-9	Tubercular strains	Growth inhibition	[11]
	25-Dien-3-one Oleanic acid (OA) Bonianic acid A Bonianic acid B	*Mycobacterium tuberculosis*	Synergistic activity alongside antibiotic	[2]
	OA Ergosterol peroxide Ursolic acid (UA)		Synergistic activity—combination therapy	
	OA UA	*B. cereus* *Vibrio cholerae* *S. choleraesuis* *K. pneumoniae* *S. pneumoniae*	Growth inhibition	[85]
		Planktonic cariogenic microorganism *S. mutans* *S. sobrinus*	Biofilm inhibition	[86] [31]
		*Listeria monocytogenes*		[45]
	OA	*A. baumanii*	Antibiotic potentiation	[4]
	Amyrin Betulinic acid Betulinaldehyde	MRSA MMSA	Growth inhibition	[87]
